# Research Progress on the Impact of Human Chorionic Gonadotropin on Reproductive Performance in Sows

**DOI:** 10.3390/ani14223266

**Published:** 2024-11-13

**Authors:** Jiahao Li, Xuedan Zhu, Wenjun Zhu, Li Li, Hengxi Wei, Shouquan Zhang

**Affiliations:** State Key Laboratory of Swine and Poultry Breeding Industry, National Engineering Research Center for Breeding Swine Industry, Guangdong Provincial Key Lab of Agroanimal Genomics and Molecular Breeding, College of Animal Science, South China Agricultural University, Guangzhou 510640, China; lijiahao@stu.scau.edu.cn (J.L.); xuedanzhu3492@163.com (X.Z.); m18672564925@163.com (W.Z.); lili007@scau.edu.cn (L.L.); weihengxi@scau.edu.cn (H.W.)

**Keywords:** human chorionic gonadotropin, sow, assisted reproduction, follicular development, corpus luteum, pregnancy

## Abstract

Human chorionic gonadotropin is a glycoprotein hormone produced by human or humanoid syncytiotrophoblasts that differentiate during pregnancy. Due to its superior stability and long-lasting effects compared to luteinizing hormone, it is often used to replace luteinizing hormone to regulate reproductive performance in sows. Human chorionic gonadotropin promotes oocyte maturation, follicle development, and luteinization, thereby increasing conception rates and supporting early embryonic development. In sow reproductive management, the application of human chorionic gonadotropin not only enhances ovulation synchrony but also improves the success rate of embryo implantation by regulating endometrial receptivity and immune mechanisms, significantly enhancing overall reproductive performance. This article primarily reviews the application of human chorionic gonadotropin in sow follicle development, luteal maintenance, and embryo implantation, providing theoretical support for its use in improving reproductive performance in sows.

## Simple Summary

Human chorionic gonadotropin is a glycoprotein hormone produced by human or humanoid syncytiotrophoblast cells differentiated during pregnancy. It functions similarly to luteinizing hormone, assisting in ovulation synchronization and supporting early pregnancy in sows by promoting follicular maturation, corpus luteum function, and embryo implantation. When administered to sows, human chorionic gonadotropin can improve conception rates, embryo implantation success, and overall reproductive performance. This review mainly summarizes the role of human chorionic gonadotropin in sow reproductive management and its application prospects.

## 1. Introduction

Chorionic gonadotropins (CGs) are glycoprotein hormones secreted by fetal-origin trophoblastic epithelial cells, including human chorionic gonadotropin (hCG) and equine chorionic gonadotropin (eCG), the latter also referred to as pregnant mare serum gonadotropin (PMSG). PMSG is a reproductive hormone commonly used in the production management of female livestock (such as pigs, cows, and sheep), administered as a single injection to meet their ovulatory and estrus requirements. Although PMSG has a luteotropic effect in pregnant mares, promoting the development of the accessory corpus luteum and contributing to pregnancy maintenance, its administration in other species results in both Luteinizing hormone (LH) and Follicle-stimulating hormone (FSH)-like effects, mainly functioning as FSH [1,2]. In addition, PMSG faces challenges such as limited sources, difficulties in extraction and purification, and risks of disease transmission [3,4]. In contrast, the hCG currently used is primarily recombinant hCG (rhCG), produced through genetic engineering. Previously, exogenous hCG was extracted from the urine of pregnant women. However, this method is inherently unstable, with significant batch-to-batch variations in biological activity, and raises concerns about the privacy of pregnant donors, making many reluctant to provide urine samples. Consequently, the exogenous hCG used in assisted reproduction today is mainly rhCG, obtained by transfecting Chinese hamster ovary cells with genes encoding the hCG α and β subunits [5]. rhCG has high purity and good stability. As a result, the use of hCG instead of PMSG has the advantage of high purity, good stability, safe and reliable sourcing, and no risk of disease transmission, and it is more suitable for the field of assisted reproduction.

hCG shares the same α subunit with LH, and their β subunits have an 80–85% sequence homology. However, unlike LH and other glycoprotein hormones, hCG can exist in various hormonal and non-endocrine forms rather than being confined to a single molecular form [6,7]. Studies have shown that treating the follicular wall with r-hLH or r-hCG in vitro produces outcomes comparable to those observed in vivo during the preovulatory LH surge. Both treatments result in the downregulation of Luteinizing hormone/choriogonadotropin Receptor (LHCGR) and Follicle-stimulating hormone Receptor (FSHR) [8]. In addition, both bind and activate LHCGR on granulosa cells and luteal cells, and activation of LHCGR is critical for hormonal function during reproduction and aids in promoting ovulation and supporting early pregnancy. Unlike LH, which has a half-life of 1–2 h, hCG has a half-life of 24 to 36 h, which helps maintain corpus luteum function and prevents premature corpus luteum regression [9,10,11]. Therefore, it is commonly believed that LH and hCG have similar biological activities and that hCG can be used to replace LH to analogize the endogenous LH surge in mammals to induce simultaneous ovulation [12].

The current review concentrates on the structural functions of hCG and their implications for luteal function, follicular development, and pregnancy promotion to date, with a view to providing a theoretical basis for the application of hCG in sow reproduction.

## 2. Structure and Function of hCG

Ascheim et al. discovered hCG in the blood and urine of pregnant women in 1927 [13]. Initially, this gonadotropic substance was believed to be produced by the anterior pituitary gland; however, Seeger-Jones et al. demonstrated that it can be produced in vitro from placental tissue cultures, concluding that it originates from the placenta [14,15]. hCG is a heterodimeric glycoprotein consisting of a common α subunit and a distinct β subunit, like FSH, LH, and thyroid-stimulating hormone. These different β subunits define their specificity [16].

hCG, produced by human or anthropoid differentiated syncytiotrophoblasts during pregnancy, serves as a crucial embryonic signal essential for maintaining pregnancy and is primarily used to identify maternal pregnancy. It has a trisaccharide structure with two N-linked sugar chains on both the α and β subunits and four O-linked sugar chains on the β subunit, with a molecular weight of 36.7 kDa, and it is secreted by placental trophoblast cells [17]. It is now common to use hCG test strips to detect urine hCG levels for pregnancy determination, but elevated hCG concentrations in non-pregnant patients may indicate cancer due to the production of hCG by tumors [18]. The β subunit of hCG contains a carboxyl-terminal peptide (CTP) structural domain at its carboxyl-terminal end, prolonging its half-life. Without the CTP, hCG’s half-life is reduced to only one-third of that of the native hCG protein, and it loses its characteristic biological activity [19].

**Figure 1:**
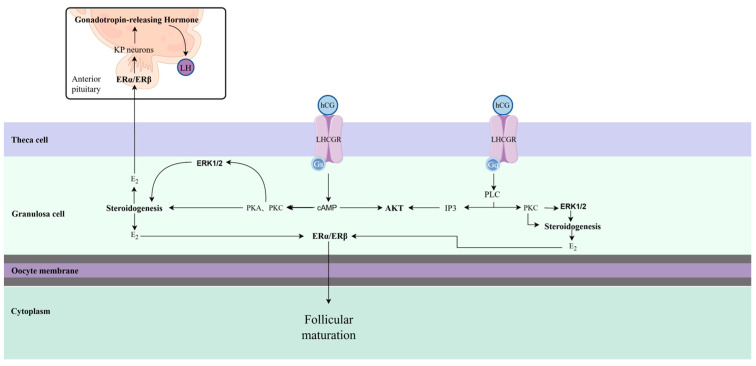
Response of hCG to LHCGR in granulosa cell (GC). Note: hCG binds to LHCGR and activates Gs and Gq proteins. cAMP or PKC and IP3 levels in granulosa cells are elevated after activation of Gs/Gq proteins, which further activates the ERK1/2 and AKT signaling pathways and promotes steroidogenesis. The generated E_2_ acts on follicles and oocytes via Estrogen Receptor α (ERα)/Estrogen Receptor β (ERβ) on the one hand, ultimately promoting follicular maturation; on the other hand, it acts on ERα/ERβ in the pituitary gland and affects Gonadotropin-releasing Hormone (GnRH) secretion. ERα/β, estrogen receptor α/β; PKA/PKC, protein kinase A/C; KP, kisspeptin; GnRH, gonadotropin-releasing hormone; IP3, Inositol phosphate 3; Gq/Gs, G protein.

When hCG interacts with LHCGR, it can activate various signaling cascades in a wide range of cell types. When hCG acts on ovarian corpus luteum cells, it can induce the production of progesterone (P_4_) by these cells, it can strengthen endometrium receptivity, and hCG also promotes angiogenesis through the upregulation of EG-VEGF [20]. Furthermore, hCG is essential in establishing pregnancy and the maternal adaptation process, as it facilitates the transition from the cyclic ovarian corpus luteum to the gestational corpus luteum [21,22]. Studies have demonstrated that hCG exerts a major favorable influence during the initial moments of embryo implantation, markedly enhancing the stability of adherent embryos [23]. hCG also improves ovum quality and enhances receptivity to pregnancy by increasing the secretion of P_4_ as well as estradiol (E_2_), which improves ovum quality and increases the fertility rate [24]. At the same time, the binding of hCG to LHCGR leads to the activation of the G-protein Gs/Gq, which, in turn, triggers the cyclic AMP (cAMP)/protein kinase A (PKA) pathway and promotes protein phosphorylation in specific cells, thereby influencing steroidogenesis [25,26] (Figure 1). This process is also influenced by the extracellularly regulated protein kinase (ERK1/2) and phosphatidylinositol 3-kinase (PI3K)/protein kinase B (AKT) pathways, with all three (cAMP/PKA, ERK1/2, and PI3K/AKT) playing roles in regulating oocytes and follicular maturation. Moreover, it has been established that LHCGR activation triggers the phospholipase C (PLC)/inositol phosphate (IP) signaling pathway, which plays a critical role in granulosa cell differentiation [27,28].

## 3. hCG Affects Follicle Maturation and Ovulation

**Figure 2:**
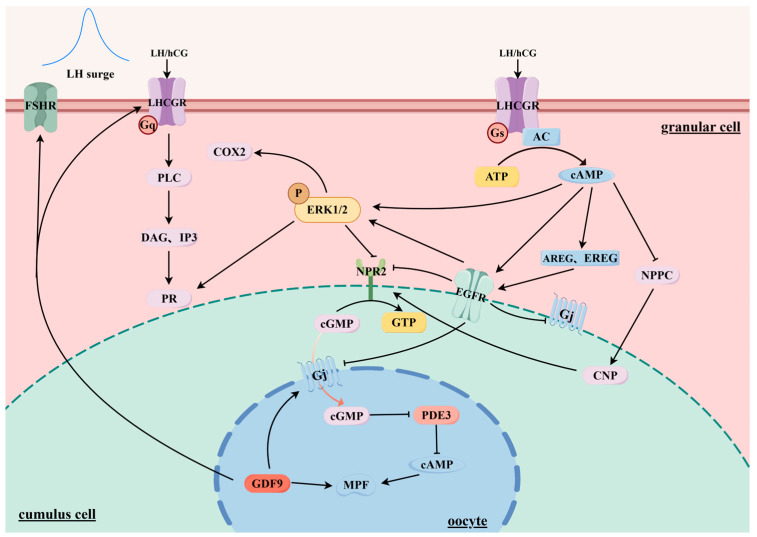
LH surge signal transduction in the ovarian follicle. Note: After binding to LHCGR, LH/hCG activates Gs protein, increasing the level of cAMP in GC. LH/hCG stimulates the expression of growth factors AREG and EREG in the GC cells. These growth factors stimulate EGFR signaling and activate the Ras-Raf-MEK pathway, resulting in the phosphorylation of ERK1/2. Progesterone receptor (PR) and COX2 expression are boosted by activated pERK1/2, which is necessary for a successful ovulation. Furthermore, NPPC mRNA and NPR2 expression are both inhibited by the cAMP and ERK1/2 pathways, respectively. Among them, NPPC mRNA mainly encodes CNP, and CNP/NPR2 can maintain the meiosis of oocytes in an arrested state. Thus, the meiotic process also resumes when cAMP and ERK1/2 suppress the production of NPR2 and NPPC mRNA. In this process, gap junction activity is also inhibited, resulting in a decrease in cGMP levels in follicles and oocytes. This inhibition activates PDE3 in the oocyte, leading to a reduction in the oocyte’s cAMP levels, which in turn activates MPF. Gq/Gs/Gj, G protein; ATP, adenosine triphosphate; cAMP, cyclic adenosine monophosphate; IP3, inositol trisphosphate; PLC, phospholipase C; ERK1/2, extracellular regulating protein kinase 1/2; AC, adenylate cyclase; PR, progesterone receptor; NPR2, natriuretic peptide receptor B; CNP, C-type natriuretic peptide; AREG, amphiregulin; EREG, epiregulin; DAG, diacylglycerol; EGFR, epidermal growth factor receptor; GTP, Guanosine-5’-triphosphate; COX2, cyclooxygenase-2; PDE3, phosphodiesterase 3; 5’-GMP, 5’-Guanosine monophosphate; MPF, Maturation-Promoting Factor; FSHR, follicle-stimulating hormone receptor; cGMP, cyclic guanosine monophosphate; GDF9, growth differentiation factor 9; NPPC, natriuretic peptide precursor C.

During physiological processes, ovarian follicles are recruited and selected by FSH during physiological processes, while their final maturity and ovulation are encouraged by LH [29]. These two gonadotropins work synergistically to support follicular development. LH binds to the endometrial cells of the antral follicles, promoting the synthesis of androgens from endogenous steroids, which further influences the granulosa cells’ response to FSH [30,31]. The LH surge triggers ovulation, and by imitating the LH surge, hCG can trigger oocyte maturation and mature follicle ovulation. Therefore, exogenous hCG is frequently employed during controlled ovarian stimulation cycles to simulate the LH surge. LH surge can trigger the regulation of oocyte meiosis recovery of signaling pathways, including cAMP and cyclic guanosine monophosphate (cGMP), epidermal growth factor receptor (EGFR), ERK1/2, maturation-promoting factor (MPF), Ca^2+^, and enzyme synthesis before ovulation (Figure 2) [32,33,34,35,36,37,38].

rhCG is clinically and statistically equivalent to urine-derived hCG (uhCG) in promoting final follicle maturation [39]. When rhCG is used to induce ovulation, it is superior to uhCG with regard to the quantity of mature oocytes obtained and partial tolerance [40,41,42]. The oocytes obtained after treatment with uhCG were less mature, which may be due to the fact that the hCG degradation products in uhCG interfere with hCG-induced oocyte maturation to some extent [43]. In addition, although the mechanism by which hCG induces ovulation does not depend on the regulation of the ovarian–pituitary axis, its action is contingent upon the existence of LHCGR on the surface of follicular granulosa cells [44]. It has been shown in pigs and cattle that during the first two days of follicle wave onset (d0 = follicle ≥ 4 mm), protein or mRNA encoding LHCGR is less expressed in granulosa cells of growing follicles [45,46,47,48,49]. Furthermore, Filicori et al. demonstrated that low-dose hCG can enhance ovarian follicle development and estrogen stimulation during ovulation induction when FSH levels remain constant or increase, significantly reducing the cost of ovulation induction procedures [50,51]. This may involve differential regulation of ovarian follicle growth by suppressing the number of small follicles [52,53,54]. On the contrary, when ovulation is induced using a Gonadotropin-releasing Hormone (GnRH) agonist (GnRH-a), its effectiveness in synchronizing ovulation within a herd of sows is limited by the number of animals with smaller follicles at the time of treatment [55,56]. Studies in humans, sheep, and cattle have shown that GnRH-a typically results in a lower implantation rate and a higher rate of ectopic pregnancies, which may be associated with reduced endometrial receptivity [57,58,59,60,61]. However, no relevant reports have been found in sows so far, and further research may be needed.

In the reproductive management of sows, assisted reproduction primarily employs timed insemination technology. The current approach involves using PMSG or FSH to stimulate follicle growth 24 h after weaning or withdrawal of progesterone, commonly referred to as estrus synchronization treatment, followed by hCG or GnRH to induce synchronous ovulation at specific intervals (the timing of hCG or GnRH administration may vary according to breed and environment) [62,63,64,65]. Intravenous or intramuscular GnRH drugs, such as buserelin and triptorelin, promote increased FSH and LH secretion [66]. However, investigations have shown that the application of GnRH is not constantly efficient in causing ovulation in sows [67,68]. On the other hand, GnRH may also have a negative impact on ovulation in weaned sows [64,69,70]. This is mainly due to GnRH’s need to work on the pituitary gland, stimulating the secretion of LH, thus forming LH surge, eventually triggering ovulation. It is evident that the action of GnRH is unstable because it mostly has an indirect effect and is reliant on the amount of LH produced by the pituitary gland. Although a particularly large LH surge is not required for ovulation, a reduced LH surge may adversely impact the quality of follicular luteinization [71]. Additionally, GnRH appears to be more likely to cause luteal phase defects in human studies. This problem may be due to the short duration of the LH surge it induces, resulting in luteal phase insufficiency, a significant decrease in steroid and non-steroid hormones, affecting endometrial receptivity, which may result in a decrease in conception rates and subsequent delivery rate decline [72,73,74]. It has also been found in sows that insufficient LH supply may result in inadequate P_4_ production by the corpus luteum, which is necessary to support proper embryonic development [75]. Additionally, studies have shown that primiparous livestock have a low response to GnRH, possibly due to insufficient endogenous LH support for follicular development [63,76,77,78]. Unlike GnRH, hCG’s physiological role is predominantly LH-like, and its mode of action operates independently of the pituitary gland. It directly mimics the endogenous LH peak in the sow by binding to LHCGR on the ovary, thus stimulating simultaneous ovulation [44,79,80]. When the follicle develops to the ovulatory stage, LHCGR expression and E_2_ production increase, and then the large follicle begins to rupture, releasing the oocytes for fertilization. Thus, hCG is less affected by the external environment and by the sow’s own pituitary LH reserves when inducing ovulation in sows. A study by Seyfang et al. showed that hCG can improve fertility in summer primiparous sows and stimulate estrogen secretion as well as follicle growth and luteal function [79]. Similar findings have been reported in dairy cows [81]. In addition, Ziecik et al. found that administering hCG to sows affects lipid metabolism, extracellular matrix remodeling, cell proliferation and survival, and protein folding, resulting in changes in key proteins such as Cholesterol side-chain cleavage enzyme, Tropomyosin-2 (TPM2), Serpin Family A Member 3 (SERPINA3), Vimentin, and Heat shock 70 kDa protein 8 [82]. The increased amounts of lipid metabolism-related proteins in the follicle wall may also indicate that the luteinization of preovulatory follicles in sows treated with hCG occurs earlier, which is beneficial to the growth of follicles. In addition, the study also showed that TPM2 in hCG treatment of sexual maturity of the gilt follicle was found to increase in the wall. TPM2 is an actin-binding protein that is necessary for the development of preovulatory follicles, including functions for cell signaling, growth, cell shape maintenance, and differentiation [83,84,85].

Sow oocytes have a lower survival duration after ovulation (about 8 h) compared to sperm after insemination (about 24 h) [86]. Therefore, ensuring that artificial insemination is performed before ovulation but near the ovulation period is key to achieving a high conception rate. According to studies, 85% to 90% of sows can trigger ovulation 42 h (39–49 h) after receiving hCG [71,87,88,89,90,91]. Gilts before puberty show obvious estrus and ovulation about 96 h after receiving PMSG and hCG treatment [92]. Synchronized estrus treatment of sows with Altrenogest followed by hCG injection at 96 h and a single Artificial Insemination (AI) 24 h later produced good conception rates [93]. Ovulation induction with hCG was performed after the injection of PMSG for 56 h or 72 h in gilts and a single AI was performed at an interval of 24 h, and the fertility rate and litter size were similar to those of the estrus check breeding group [94]. For weaned sows not pre-injected with PMSG, 750 IU of hCG was injected at 80 h post-weaning, and the farrowing rates increased by 15% relative to the control group [95]. Sows were injected with 1000 IU of PMSG 24 h after weaning and 500 IU of hCG 72 h or 56 h after treatment, followed by an AI 24 h later. There were significantly elevated piglets compared to the control group [96,97]. Belstra et al. showed that sows that underwent a single AI at 17–24 h before ovulation had a higher pregnancy rate [98]. A single AI performed 24 to 33 h after hCG treatment of weaned sows resulted in delivery rates and litter sizes comparable to those achieved with multiple inseminations [71]. Obviously, using hCG to synchronize ovulation after weaned sows are in estrus and performing single insemination about 24 h after hCG injection can also increase the likelihood of conception and litter size to an equivalent degree as multiple inseminations. However, due to the different pig herd conditions and farm environments in different pig farms, the time interval between estrus and hCG injection may vary. The specific time of insemination after hCG injection should also be determined according to the specific situation.

Overall, the LH surge is key to ovulation. Exogenous hCG mimics the LH surge, accelerates oocyte maturation, and is progressively replacing uhCG with rhCG due to its superior consistency and purity. Both GnRH and hCG are used to time insemination and induce ovulation, but the effect of GnRH is unstable and is affected by environmental factors and pituitary LH reserves, while hCG is more stable and is effective by acting directly on the ovary. Timely insemination after synchronized estrus and ovulation significantly increases conception rates, and by optimizing the AI procedure, the reproductive efficiency of sows can be effectively improved.

## 4. hCG Supports Luteal Function

In the aftermath of ovulation, the ruptured follicle forms a corpus hemorrhagic and then rapidly transforms into a vascularized gland-like structure called the corpus luteum. The corpus luteum degenerates into the corpus albicans, which declines and initiates an estrous cycle if the discharged ovum is not fertilized. In the event that the ovum is fertilized, luteinizing hormones stimulate the change of the functional corpus luteum into the gestational corpus luteum, which supports pregnancy [99]. The corpus luteum of primates induces significant quantities of P_4_, as well as moderate amounts of E_2_; the corpus luteum in domestic animals and rodents produces only P_4_, which is essential for the formation and maintenance of pregnancy [100].

hCG can improve the corpus luteum’s survival and steroidogenic function by binding to LHCGR on luteinizing cells and GCs. In pregnant gilts administered 750 IU of hCG, increased expression of steroidogenic acute regulatory protein (STAR) and LHCGR was observed, accompanied by enhanced angiogenesis, reduced apoptosis of corpus luteum cells, and an increase in viable cells [101,102]. Evidence from studies on cows and sheep indicates that hCG can improve the function of the primary corpus luteum, induce the formation of auxiliary corpus luteum, and increase conception rates [103,104]. Administering additional hCG to sows can also promote follicular luteinization and enhance corpus luteum function [105]. In addition, studies in pigs and cattle have shown that low circulating P_4_ concentrations may negatively impact pregnancy maintenance in females [106,107,108]. However, hCG can increase the luteal synthesis of P_4_ via the JNK signaling pathway [109].

Premature luteal regression is a common indication of luteal insufficiency and one of the causes of embryonic death, which may be related to abnormal secretion of uterine prostaglandin-F_2α_ (PGF_2α_) [110,111,112]. It was reported by Dias et al. that hCG treatment reduced structural degeneration of the corpus luteum in ewes by around threefold [113]. In addition, experiments on pigs and cattle have found that hCG’s effect in preventing premature regression of the corpus luteum may be achieved through an LH-like effect that converts small corpus luteum cells into large corpus luteum cells, promoting steroid production in the corpus luteum and increasing P_4_ levels [114,115]. It has been demonstrated that the application of hCG in cattle promotes early embryonic development by aiding in the establishment of an auxiliary corpus luteum and increasing the amount of luteal tissue [116,117]. Sows can effectively extend the longevity of the corpus luteum with a single injection of hCG on day 12 of the estrous cycle, as demonstrated by Guthrie et al. [118]. This may be achieved by hCG through elevating the Prostaglandin E_2_ (PGE_2_)/PGF_2α_ ratio and circulating E_2_ levels [102,119,120]. It has been shown that PGE_2_ biosynthesis, transport, and signaling pathways are selectively activated during luteal maintenance, with the PGF_2α_ system being engaged during luteal decline [121]. During sow pregnancy, E_2_ triggers maternal recognition and maintains luteal function [122]. Furthermore, hCG enhances the function of the gestational corpus luteum in sows by promoting the expression of LHCGR and STAR mRNA [102,123].

## 5. The Maintenance of Pregnancy by hCG

Pregnancy is usually defined as the process of conception and growth of an embryo or fetus in a female animal and mainly includes the processes of fertilization, embryo implantation, the entire development and growth of the embryo in the uterus, and delivery. In mono-ovulatory animals, such as humans, the oocyte is fertilized in the fallopian tube, develops into the morula stage, and then enters the uterine cavity, where it continues developing into a blastocyst and begins implantation in the maternal endometrium, whereas in polyovulatory species, such as pigs, the embryo enters the uterus at the four-cell stage, migrates throughout the uterine cavity as it develops into a blastocyst, and implants approximately 14 to 18 days after ovulation [124,125,126]. Successful implantation is one of the prerequisites for a successful pregnancy. Statistically, 20–30% of abortions in sows are attributed to implantation failure, and the proportion can reach 70% during embryo transfer [106,127]. In ruminants, implantation failure leads to abortion in 50–75% of cases [128]. In humans, the proportion is 78% [129]. Embryo implantation is defined as the establishment of a connection between trophoblast cells and endometrial cells, which allows the embryo to attach to the luminal surface of the endometrium, migrate through the luminal epithelium, and enter the depth of the endometrium. Inadequate uterine tolerance, an overactive immune system, and defective blastocyst attachment are the reasons for the failure of implantation [130,131]. Successful embryo implantation requires precise synchronization between the mother’s uterus and the developing embryo, which is known as the implantation window [132]. This window is primarily defined by a combination regulated by P_4_ and E_2_, which includes local cytokines and growth factors, glycoproteins on the surface of cells, adhesion molecules, and proteins of the extracellular matrix [133]. In sows, the implantation window mainly refers to the period from day 12 to day 30 after mating; in cows, it is from day 13 to day 16 of pregnancy; in ewes, it is from day 14 to day 21 of pregnancy; and in fertile human females, it refers to the period approximately 6 to 10 days after ovulation [134,135,136,137,138,139]. In contrast, hCG is a well-recognized molecule with pregnancy-maintaining effects, which can act on the endometrium to regulate its tolerance and also contribute to embryo implantation by mediating immunomodulation.

### 5.1. hCG Affects Endometrial Receptivity

**Figure 3:**
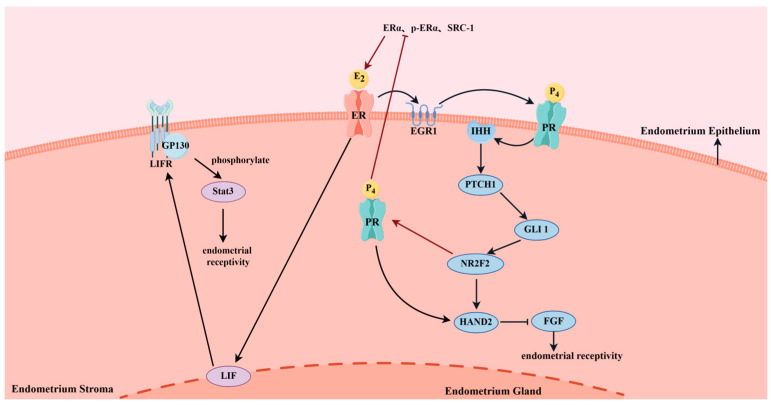
The coordinated action of E_2_ and P_4_ with their own receptors affects endometrial receptivity. Note: On the one hand, E_2_ binding to ER stimulates the production of LIF and then phosphorylates STAT3 through LIFR and its co-receptor GP130 in the endometrial epithelium, thereby affecting endometrial receptivity. On the other hand, E_2_ binding to ER can regulate EGR1, thereby maintaining PR activation and promoting P_4_ signaling. P_4_ binds to PR to activate IHH, and IHH binds to its receptor PTCH1 to activate Gli1, further affecting NR2F2 and HAND2. HAND2 can inhibit the expression of FGF, thereby affecting endometrial receptivity. When P_4_ binds to PR in endometrial stromal cells, it can affect the expression of HAND2, while NR2F2 can mediate the inhibition of estrogen activity in the endometrial epithelium by inhibiting the expression of SRC-1 and ERα and the activation of ERα through PR in the endometrial stroma. HAND2, Heart And Neural Crest Derivatives Expressed 2; EGR1, early growth response proteins1; PR, progesterone receptor; ERα, estrogen receptor α; SRC-1, Steroid Receptor Coactivator 1; E_2_, estrogen; P_4_, progesterone; LIFR, leukemia inhibitory factor receptor; LIF, Leukemia inhibitory factor; FGF, Fibroblast growth factor; NR2F2, Nuclear Receptor Subfamily 2 Group F member 2; GLI 1, Zinc finger protein GLI1; PTCH1, Protein Patched Homolog 1; NR2F2, Nuclear Receptor Subfamily 2 Group F member 2; IHH, Indian Hedgehog; GP130, Signal Transducing Subunit gp130; ER, estrogen receptor; LIFR, leukemia inhibitory factor receptor; STAT3, Signal transducer and activator of transcription 3.

Embryo implantation in domestic animals is largely dependent on the function of the endometrium. The basic molecular mechanisms of embryo implantation are relatively conserved among mammalian species. Studies have shown that E_2_ and P_4_ secreted by the ovaries affect endometrial receptivity through the coordinated action of autoreceptors located in the endometrial epithelium [140,141,142] (Figure 3). However, the times at which E_2_ and P_4_ play dominant roles are not consistent. During the implantation period, P_4_ takes the lead, while E_2_ is dominant before implantation. It is crucial for endometrium receptivity that the levels of E_2_ are boosted prior to implantation. Previous experiments on mice have found that E_2_ can regulate early growth responsive gene-1 (EGR1) in the endometrial epithelium and stroma through ER [143]. EGR1 plays a vital role in maintaining PR activation and promoting P_4_ signal transduction [144]. Studies on mice have found that during this process, E_2_ can also stimulate the production of Leukemia Inhibitory Factor (LIF), which in turn phosphorylates Signal Transducer and Activator of Transcription 3 (STAT3) through LIFR and its coreceptor signal transduction subunit gp130 (GP130) in the endometrial epithelium, thereby affecting the embryo implantation process [145,146,147]. Studies in sows have shown that hCG promotes E_2_ production [8]. Some studies also discovered that porcine oocytes cultured in vitro with hCG significantly elevated EGR1 expression [148,149]. However, it is unknown if hCG injections can influence the expression of related genes in the porcine endometrium before implantation. On the other hand, studies on mice have also found that P_4_ can bind to PR in the endometrial epithelium and activate the Indian Hedgehog homolog (IHH) gene in the endometrial epithelium [150]. IHH then binds to its target gene Protein Patched Homolog 1 (PTCH1) and activates Gli1 in endometrial stromal cells [151,152]. Furthermore, it affects the transcription factor of the Nuclear Receptor Subfamily 2 Group F member 2 (NR2F2) and the Heart And Neural Crest Derivatives Expressed 2 (HAND2), thereby preparing for embryo implantation in the endometrium [153,154,155,156,157]. In addition, when P_4_ binds to PR in endometrial stromal cells, it can affect the expression of HAND2, while NR2F2 can inhibit the expression of steroid receptor coactivator 1 (SRC-1) and ERα and the activation of ERα through PR in the endometrial stroma [144,154]. This mediation leads to the inhibition of endometrial epithelial estrogen activity, which is important for creating a receptive uterus. At present, reports on pigs, cattle, and sheep have found that hCG can directly act on the corpus luteum; reduce the number of small corpora lutea; increase the number of large luteal cells; and enlarge the diameter, surface area, and volume of the corpus luteum, thereby increasing P_4_ production and regulating endometrial receptivity [114,158,159,160,161,162,163,164,165]. In addition, hCG administration at a specific time after AI in these species can stimulate corpus luteum function, increase progesterone concentration, and reduce estrogen production, thereby positively affecting pregnancy rates and embryo survival [79,102,166,167,168,169].

Angiogenesis plays a key role in endometrial maturation. As early as 1986, Ziecik et al. confirmed the existence of LHCGR in extragonadal tissues such as the porcine myometrium and that there is a high affinity between the receptors and ligands in these tissues [170]. For example, the number of uterine LH binding sites in the myometrium is 10 times the receptor capacity of the pig corpus luteum. Studies have revealed that the use of hCG to treat porcine endometrial tissue promotes angiogenesis, which may be due to its affecting the secretion of vascular endothelial growth factor (VEGF) [102,171,172]. VEGF is a heparin-binding glycoprotein that is essential for enhancing vascular permeability, stimulating endothelial cell proliferation, differentiation, migration, and capillary assembly, and it can synergistically regulate vascular development and function with angiopoietin. The literature has shown that increased P_4_ during early pregnancy can stimulate VEGFA expression in the endometrium [79,173]. Therefore, the use of hCG in early pregnancy may promote angiogenesis by affecting P_4_ secretion, thereby preparing the endometrium for embryo implantation. hCG can also regulate the ratio of PGE_2_:13,14-dihydro-15-keto-PGF_2α_ (PGFM) throughout the gestational cycle and affect the development of the embryo by promoting angiogenesis while reducing corpus luteum apoptosis in pre-pregnant pigs [102]. Furthermore, researchers have demonstrated in cattle and sows that P_4_ can downregulate the expression of MUC1, a highly glycosylated transmembrane mucin glycoprotein that inhibits embryo implantation through steric hindrance [142,174,175,176]. hCG can promote the secretion of P_4_, so hCG may promote embryo implantation by promoting the production of P_4_, thereby inhibiting the expression of MUC1.

Furthermore, Ziecik et al. found that SERPINA3 was upregulated in the follicular wall of hCG-treated sexually mature reserve sows [82]. SERPINA3, often referred to as α 1-antichymotrypsin, is a member of the acute-phase protein and serine protease inhibitor group, which triggers inflammatory cytokines and remodels tissues [177]. Research on baboons has demonstrated that hCG-induced expression of SERPINA3 in the endometrial stroma contributes to cellular remodeling at the site of embryo implantation, but this has not been reported in sows [178]. Zlotkowska et al. demonstrated that the chemokine CXCL12, which is generated by the endometria and trophoblasts of sows, contributes to the endometrial preparation of embryos for implantation by regulating endometrial tolerance and facilitating embryo adhesion [179]. In addition, hCG upregulates CXCL12 expression, further supporting embryo attachment [180]. When hCG was applied to pig endometrial epithelial cells, Hwangbo et al. observed an increase in plasminogen activator (PA) activity. This resulted in the remodeling of the uterine tissue and the modulation of the intrauterine environment, both of which supported embryonic survival [181]. The PA system is essential for tissue remodeling throughout the estrous cycle and embryo implantation and influences processes such as angiogenesis, secretory gland function, and endometrial thickness [181,182]. Furthermore, it has been proposed that the chemokine CCL8 has a role in the onset of pregnancy in sows; however, it has not been shown whether hCG influences CCL8 expression. Additionally, it has also been demonstrated that porcine endometrial lymphocytes are crucial for angiogenesis by transcribing and translating vascular endothelial and placental growth factors, as well as their receptors, kinase insertion domain receptor (KDR) and fms-associated tyrosine kinase 1 (FLT1); these cells also regulate angiogenesis during healthy pregnancy by expressing Hypoxia-inducible factor-1α (HIF1A) [183]. However, it has not been reported whether hCG can regulate angiogenesis during pregnancy by acting on porcine endometrial lymphocytes and thereby affecting the above factors.

Clearly, embryo implantation is dependent on endometrial function, which is coordinately regulated by E_2_ and P_4_. hCG can affect the secretion of E_2_ and P_4_, thereby affecting endometrial receptivity and promoting embryo implantation. In addition, hCG supports uterine remodeling and embryo attachment by regulating factors such as VEGF, SERPINA3, and CXCL12. hCG also regulates the PGE_2_:PGFM ratio during the estrous cycle, promotes angiogenesis, and reduces corpus luteum apoptosis, thereby affecting the development of the embryo. Although the role of hCG in regulating endometrial receptivity has been well studied, the specific mechanisms underlying its effects on these factors require further investigation.

### 5.2. hCG-Mediated Immunomodulation Contributes to Embryo Implantation

From an immunological standpoint, pregnancy involves a subtle immunity paradox in which the woman and the embryo must be protected by an effective immune response, yet immune tolerance is necessary to prevent embryo rejection, facilitate implantation, and advance the pregnancy [184]. The immune cells which are at the maternal–fetal interface, including T cells, macrophages, and NK cells, are essential for placental development, fetal tolerance, and the preservation of physiological homeostasis [185,186,187], along with their associated cytokines, which impact embryo implantation and development, including interleukin (IL), Tumor Necrosis Factor-α (TNF-α), and Interferon (IFN), among others [188,189,190,191].

Porcine conceptuses produce IL-1β during early development and elongation [192,193]. On days 10 to 13 of pregnancy, IL-1β activates the expression of microsomal prostaglandin E synthase-1 mRNA in uterine tissue, stimulating the synthesis and secretion of PGE_2_ [194,195,196]. PGE_2_ can influence the production of granulocyte-macrophage colony-stimulating factor, which is produced by the fallopian tube and endometrium and acts on preimplantation embryos to enhance their ability to establish pregnancy [197,198]. Clearly, IL-1β influences the expression of favorable cytokines at the maternal–fetal interface, mediating uterine regulatory mechanisms in early pregnancy. In addition, studies indicate that during embryo implantation, E_2_ and IL-1β stimulate the expression of endometrial IFN signaling molecules, synergistically activating IFNG and IFND produced by the pregnant body to regulate the immune response of the endometrium in early pregnancy [134]. Recent studies have shown that hCG can affect E_2_ secretion in sows during early pregnancy, but its effect on IL-1β has only been studied in humans, and there is no evidence of its effect on IL-1β production in sows [8,199]. IL-6 is a multifunctional cytokine that plays a crucial role in various aspects of placental development during early pregnancy [195]. Studies have found that IL-6 shows significant changes on the 12th and 14th days of pregnancy in sows, suggesting that IL-6 is crucial for embryo–maternal crosstalk during sow embryo implantation [200,201]. Additionally, studies have shown that after hCG culture of bovine endometrial cells in vitro, IL-6 mRNA expression is inhibited, reducing immune rejection and facilitating embryo implantation [202]. However, there are no reports on whether hCG affects IL-6 expression in sows and promotes embryo implantation. Macrophage migration inhibitory factor (MIF) has potent pro-angiogenic properties and immunomodulatory effects, especially in regulating macrophage migration and inhibiting NK cell activity [203]. Studies have shown that MIF determines the aggregation and activation of macrophages at the maternal–fetal interface through autocrine/paracrine secretion during early placental formation in sows [204,205,206]. In humans, hCG can directly stimulate endometrial stromal cells to produce and release MIF, but there are no reports on whether hCG can regulate the expression of MIF in sows. Obviously, hCG has a significant positive effect on immunosuppression during pregnancy, but current research on its immunosuppressive effects during embryo implantation in sows remains limited. Although the basic molecular mechanisms of mammalian embryo implantation are relatively conserved, differences in placental types and structures among animals may result in differences in hCG-mediated immune regulatory mechanisms [207,208].

In summary, hCG plays an important role in placental development and immune tolerance by regulating immune cells and cytokines. However, due to differences in placental types and structures among animals, as well as limited research on its immunosuppressive effects during embryo implantation in sows, further research is needed on the immune regulatory mechanism of hCG during embryo implantation in sows to enhance its application in sow reproduction.

## 6. Conclusions

hCG is widely used in sow reproduction, acting on different cell types through various signaling pathways to promote corpus luteum formation and function, extend corpus luteum lifespan, support follicular development and maturation, increase the number of dominant follicles, and improve conception rates. Additionally, hCG facilitates embryo implantation, angiogenesis, and immune regulation during pregnancy. However, its mechanisms vary between livestock species and warrant further investigation—particularly its application to immunomodulation during embryo implantation in sows. In summary, this article outlines the key role and mechanisms of human chorionic gonadotropin in enhancing reproductive performance in sows and provides a theoretical framework for its application and optimization in actual production.
